# The complete chloroplast genome sequence of *Ehretia dicksonii* Hance (Ehretiaceae)

**DOI:** 10.1080/23802359.2022.2061873

**Published:** 2022-04-14

**Authors:** Xiaogang Xu, Yao Cheng, Lili Tong, Lu Tian, Chongli Xia

**Affiliations:** a Co-Innovation Center for Sustainable Forestry in Southern China, College of Biology and the Environment, Key Laboratory of State Forestry and Grassland Administration on Subtropical Forest Biodiversity Conservation, Nanjing Forestry University, Nanjing, China; b State Environmental Protection Scientific Observation and Research Station for Ecology and Environment of Wuyi Mountains, Nanping, China; c School of Horticulture & Landscape Architecture, Jinling Institute of Technology, Nanjing, China

**Keywords:** *Ehretia dicksonii*, complete chloroplast genome, phylogenomics, Ehretiaceae

## Abstract

*Ehretia dicksonii*_Hance 1862 is a deciduous botany of Ehretiaceae. In this work, the complete chloroplast (cp) genome sequence of *E*. *dicksonii* was probed by next generation sequencing in an effort to provide genomic resources useful for promoting its conservation. The complete cp genome of *E*. *dicksonii* is 156,623 bp in length, including a large single-copy (LSC) region of 86,853 bp, and a small single-copy (SSC) region of 18,150 bp. It contains 133 genes, including 37 tRNA genes, 8 rRNA genes, and 88 protein-coding genes. The overall GC content of *E*. *dicksonii* chloroplast genome is 37.85%. The phylogenetic analysis suggests that *E*. *dicksonii* is in the clade of Ehretiaceae other than Boraginaceae. Also, *E*. *dicksonii* has a close relationship with *Ehretia acuminata*_Brown 1810 in Ehretiaceae.

*Ehretia dicksonii* is a deciduous botany of the family Ehretiaceae. It is about 15 m high, 20 cm in diameter at breast height; mainly distributing in dense forests of the fertile soil at the foot of the mountain at an altitude around 125–600 m in Southwest, South and East of China. *E*. *dicksonii* is valued for its ecological and medicinal purposes, its bark can be used as raw materials for subsidence of a swelling (Liu and Wu 2013). The leaf blade of *E*. *dicksonii* abaxially densely and minutely hispid, hairs discoid at base, extremely scabrous, adaxially densely pubescent. It is an appropriative tree species for urban greening and border tree, especially for dust retaining (Wang et al. 1989; Zhu et al. 2003). However, there has been little progress on its complete chloroplast genome for clarifying its taxonomic status. The objective of this work was to explore the intrinsic distinction and in an effort to prove its taxonomic status in genus *Ehretia*. Also, to reveal the complete genome sequence of *E*. *dicksonii* could play an important role in the protection, development and utilization of its resources.

The fresh leaves of *E*. *dicksonii* were collected from the campus of Nanjing Forestry University, Jiangsu Province (E 118° 48′ 33", N 32° 4′ 45") in China. A specimen was deposited at Nanjing Forestry University (contact person: Xuehong Ma; email: xuehongma@njfu.edu.cn) under the voucher number NF2021040. According to the International Union for Conservation of Nature (IUCN) policy on endangered species research, the sample collection and the study was conducted with permission from Arboretum of Nanjing Forestry University. Then total genomic DNA was extracted with Plant DNA Kit (Genepioneer Biotechnologies, Nanjing, China). The complete cp genome sequence of *E. dicksonii* (GeneBank accession number: MZ555766) was characterized based on Illumina pairend sequencing data to provide a valuable complete cp genomic resource. The DNA fragments were passivated, repaired and bonded by ultrasonic wave and selected by agarose gel electrophoresis. The sample of genome sequencing library was formed by PCR amplification, which was carried out on Illumina Novaseq platform by Nanjing Genepioneer Biotechnologies Inc. (Nanjing, China), and read long for PE150 sequencing.

The original reading was filtered by fastp (version 0.20.0), and the clean data were assembled into chloroplast genome using SPAdes (Bankevich et al. 2012). Next, the reference sequence (Genebank accession number: MF179500.1) was used for quality control after assembly. Finally, the assembled genome was annotated using CpGAVAS (Liu et al. 2012).

The complete chloroplast genome sequence of *E*. *dicksonii* was 156,623 bp in length. The genome had a typical quadripartite structure including a pair of IR (IRa and IRb) regions of 25,810 bp that were separated by an LSC region of 86,853 bp and a SSC region of 18,150 bp. A total of 133 genes were encoded, including 8 rRNA genes (4 rRNA species), 37 tRNA genes (28 tRNA species), and 88 protein-coding genes (80 CDS species). Most of the genes occurred in a single copy; however, 8 protein-coding genes (*ndhB*, *rp12*, *rp123*, *rps12*, *rps7*, *ycf1*, *ycf*15 and *ycf2*), 9 tRNA genes (*trnA*-*UGC*, *trnG*-*GCC*, *trnI*-*CAU*, *trnI*-*GAU*, *trnL*-*CAA*, *trnM*-*CAU*, *trnN*-*GUU*, *trnR*-*ACG* and *trnV*-*GAC*), and 4 distinct rRNA genes (*23S, 16S, 5S* and *4.5S*) are duplicated. A total of 10 protein-coding genes (*atpF*, *ndhA*, *ndhB*, *petB*, *petD*, *rpl16*, *rpl2*, *rpoC1*, *rps12*, *rps16*) contained 1 intron while the other 2 genes (*clpP*, *ycf3*) had 2 introns each. The overall GC content of the chloroplast genome is 37.85%. In addition, the GC contents of the LSC, SSC and IR regions are 35.87%, 32.2% and 43.15%, respectively.

To reveal the phylogenetic evolution of *E*. *dicksonii*, the phylogenetic tree (phylogram) was constructed based on 4 cp genomes from Ehretiaceae, 7 cp genomes from Boraginaceae and 1 cp genome from Carlemanniaceae as outgroups.

After sequence aligment by MAFFT (Rozewicki et al. 2019), IQTREE (Gao et al. 2018) was used to perform maximum Likelihood (ML) tree with the TVM + F+R3 model. The bootstrap method was used to test the reliability of phylogeny with 1000 replicates. The phylogenetic analysis result supported that Ehretiaceae and Boraginaceae are in 2 clades and belong to distinct taxa, *E*. *dicksonii* and *E*. *acuminata* are sister species, and they are in relative late differentiation stage in the clade of Ehretiaceae (Figure 1). As *E*. *dicksonii* had a close relationship with *E*. *acuminata* in Ehretiaceae, it can be inferred that *E*. *dicksonii* probably contains similar natural products with *E*. *acuminata* (Li et al. 2010), which can promote the study of its resource value, which needs to be further studied.

**Figure 1.**
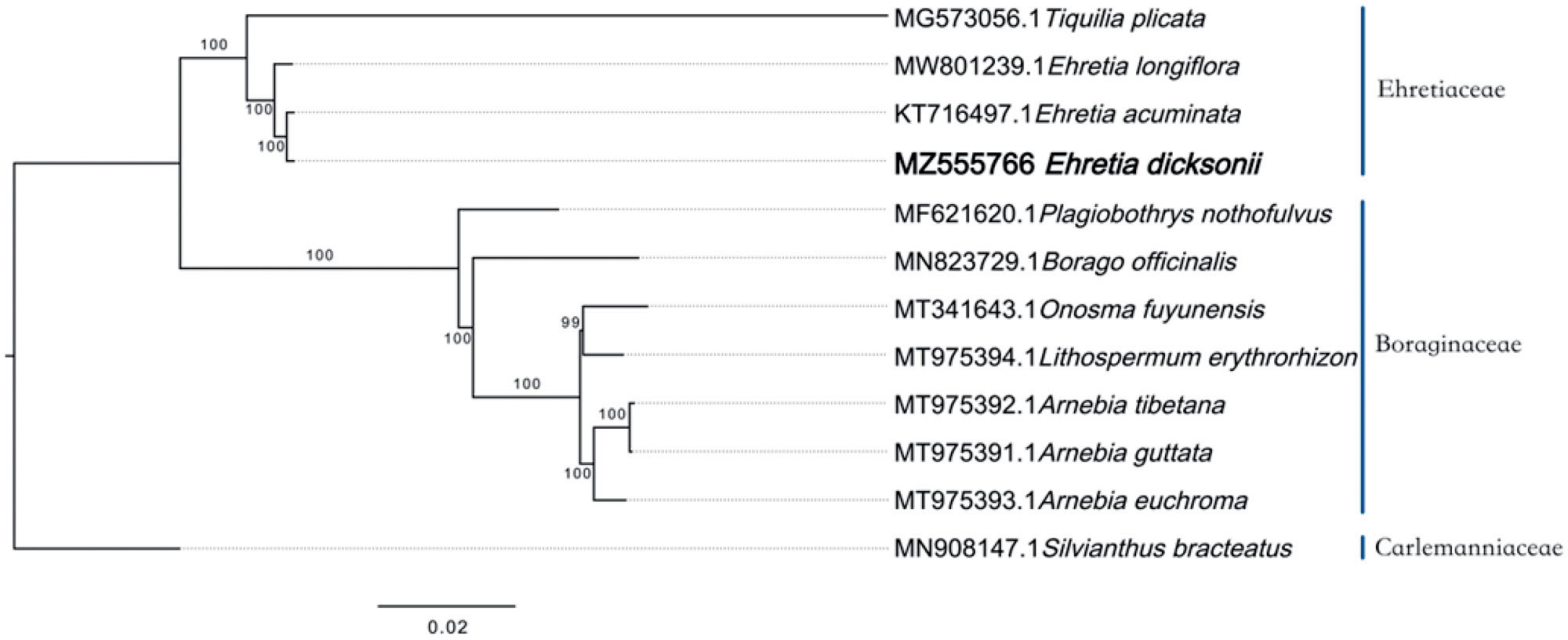
A maximum-likelihood tree was constructed based on the chloroplast genomes of 12 species. 8 species were used as outgroups from 2 taxa (Carlemanniaceae and Boraginaceae). The bootstrap supported the values shown at the branches.

## Data Availability

The genome sequence data that support the findings of this study are openly available in GenBank of NCBI at [https://www.ncbi.nlm.nih.gov] (https://www.ncbi.nlm.nih.gov/) under the accession no. MZ555766. The associated BioProject, SRA, and Bio-Sample numbers are PRJNA745580, SRR15100910, and SAMN20169761 respectively.
